# Peptide selectivity discriminates NK cells from KIR2DL2‐ and KIR2DL3‐positive individuals

**DOI:** 10.1002/eji.201444613

**Published:** 2014-11-29

**Authors:** Sorcha Cassidy, Sayak Mukherjee, Thet Mon Myint, Berenice Mbiribindi, Helen North, James Traherne, Arend Mulder, Frans HJ Claas, Marco A Purbhoo, Jayajit Das, Salim I Khakoo

**Affiliations:** ^1^ Clinical and Experimental SciencesFaculty of MedicineUniversity of SouthamptonSouthampton General HospitalSouthamptonUK; ^2^ Division of MedicineImperial College LondonLondonUK; ^3^ Battelle Center for Mathematical MedicineThe Research Institute at the Nationwide Children's HospitalColumbusOHUSA; ^4^ NHSBT ColindaleLondonUK; ^5^ Department of PathologyUniversity of CambridgeCambridgeUK; ^6^ Department of Immunohaematology and Blood TransfusionLeiden University Medical CenterLeidenThe Netherlands; ^7^ Department of Pediatrics and Department of PhysicsThe Ohio State UniversityColumbusOHUSA

**Keywords:** Killer‐cell immunoglobulin‐like receptors, MHC class I, Natural killer cells, Peptide, Peptide selectivity

## Abstract

Natural killer cells are controlled by peptide selective inhibitory receptors for MHC class I, including the killer cell immunoglobulin‐like receptors (KIRs). Despite having similar ligands, KIR2DL2 and KIR2DL3 confer different levels of protection to infectious disease. To investigate how changes in peptide repertoire may differentially affect NK cell reactivity, NK cells from KIR2DL2 and KIR2DL3 homozygous donors were tested for activity against different combinations of strong inhibitory (VAPWNSFAL), weak inhibitory (VAPWNSRAL), and antagonist peptide (VAPWNSDAL). KIR2DL3‐positive NK cells were more sensitive to changes in the peptide content of MHC class I than KIR2DL2‐positive NK cells. These differences were observed for the weakly inhibitory peptide VAPWNSRAL in single peptide and double peptide experiments (*p* < 0.01 and *p* < 0.03, respectively). More significant differences were observed in experiments using all three peptides (*p* < 0.0001). Mathematical modeling of the experimental data demonstrated that VAPWNSRAL was dominant over VAPWNSFAL in distinguishing KIR2DL3‐ from KIR2DL2‐positive donors. Donors with different KIR genotypes have different responses to changes in the peptide bound by MHC class I. Differences in the response to the peptide content of MHC class I may be one mechanism underlying the protective effects of different KIR genes against infectious disease.

## Introduction

NK cells are innate lymphocytes involved in the immune response to viruses and cancer, either through direct interaction with target cells or through interactions with macrophages, dendritic cells, and T cells 1, 2. They express activating and inhibitory receptors on the cell surface and signals transduced from these receptors are integrated to determine whether or not an individual NK cell is activated 3. While the activating receptors are derived from a number of different gene families and have diverse ligands, the dominant inhibitory receptors expressed on NK cells are either from the killer cell immunoglobulin‐like receptor (KIR) family or the C‐type lectin‐like receptor CD94:NKG2A and have MHC class I ligands 4, 5. Thus NK cell responses can be fine‐tuned by interactions with many different ligands including MHC class I.

A number of immunogenetic studies have highlighted the contribution that the KIR/MHC system makes to human disease including viral infections, pregnancy‐associated disorders, autoimmune disease, and cancer 6, 7, 8. The HLA‐C specific KIR are implicated in the immune response to diseases such as HCV, HIV, malaria, and psoriatic arthropathy 9, 10, 11, 12, 13, 14. For instance, in HCV infection, KIR2DL3 in combination with its HLA‐C group 1 ligands is protective, whereas KIR2DL2, which also has group 1 HLA‐C allotypes as ligands, is not 15, 16, 17. Binding studies have shown that KIR2DL2 is a higher affinity receptor for group 1 HLA‐C allotypes than KIR2DL3, suggesting that KIR2DL3‐positive NK cells are more easily activated than their KIR2DL2‐positive counterparts. However, in functional experiments, no difference has been observed in the inhibition of KIR2DL2‐ and KIR2DL3‐positive NK cells by group 1 HLA‐C alleles 18, 19. Binding of both KIR2DL2 and KIR2DL3 to MHC class I is dependent on the peptide bound by HLA‐C 20, 21, 22. However, this is a broad specificity defined by residues 7 and 8 of the bound peptide as distinct from the fine specificity of the T‐cell receptor. Recent work has demonstrated an unexpected sensitivity of NK cells to combinations of peptides presented by MHC Class I 23, 24. Thus, when the peptide VAPWNSDAL is presented by HLA‐Cw*0102 it does not alter the reactivity of KIR2DL2‐ and KIR2DL3‐positive NK cells. However, it can significantly reduce the inhibition due to a strong inhibitory peptide VAPWNSFAL in a process termed “peptide antagonism.” This implies that NK cells may be sensitive to changes in peptide repertoire and, based on the immunogenetic findings, suggests that KIR2DL2‐ and KIR2DL3‐positive NK cells may respond differently to these changes.

We originally screened a panel of P7 and P8 variants of VAPWNSLSL, a naturally processed HLA‐Cw*0102 epitope 23, 25. We identified three classes of peptides, strong inhibitory peptides, weak inhibitory peptides, and noninhibitory/antagonist peptides 23. We postulate that these represent the major classes of peptides presented by MHC class I that can influence NK cell reactivity. We have used peptides representative of these different categories to investigate how NK cells from donors with different genetic backgrounds (KIR2DL3 homozygotes and KIR2DL2 homozygotes) may respond to changes in the peptide content of MHC class I.

## Results

### A weakly inhibitory peptide discriminates KIR2DL2‐poisitve NK cells from KIR2DL3‐positive NK cells

**Figure 1:**
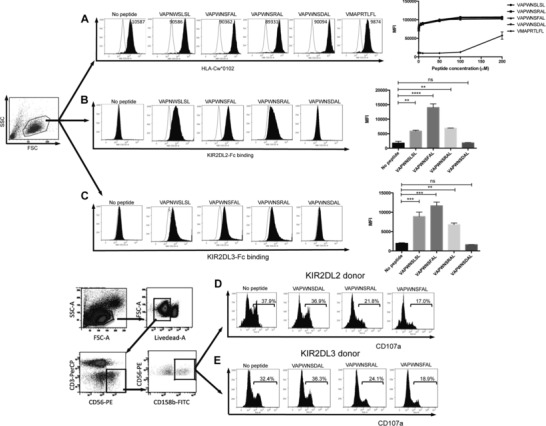
Correlation of KIR binding with NK cell inhibition in KIR2DL2+ and KIR2DL3+ donors. (A) Stabilization of HLA‐Cw*0102 on 721.174 by the indicated VAPWNSLSL derivative peptides and a control peptide (VMAPRTLFL) at saturating concentrations 10 μM (filled histograms). The no primary Ab control is indicated in each plot and the titration of the peptides is shown in the far right panel. Binding of (B) KIR2DL2 or (C) KIR2DL3 fusion constructs to 721.174 cells in the presence (filled lines) or absence of the indicated peptide at a concentration of 10 μM. Representative histograms (*n* = 3 samples) are shown. Summaries of the mean fluorescence intensities (MFI) of KIR‐binding pooled from two independent experiments are shown in the far right panels (**p < 0.01, ***p < 0.001; Student's *t‐*test). Gating strategy and degranulation assays of CD158b‐positive CD3‐CD56+ NK cells from (D) KIR2DL2 homozygous or (E) KIR2DL3 homozygous donors in response to 721.174 cells incubated with the indicated peptides at a final concentration of 10 μM. Representative histograms of six 2DL2 and eight 2DL3 donors, analyzed in duplicate, are shown.

**Figure 2:**
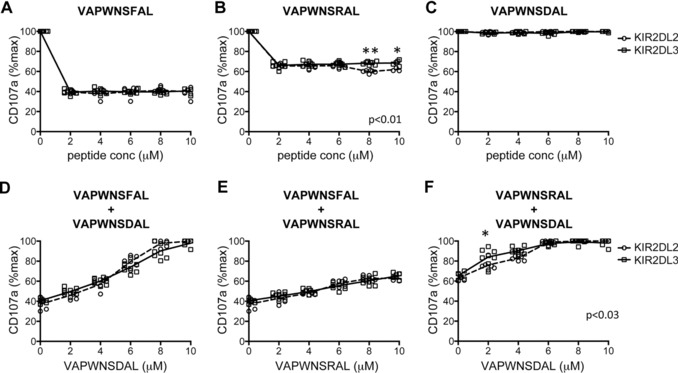
Comparison of inhibition of CD158b+ NK cells from KIR2DL2+ and KIR2DL3+ donors by individual peptides and double peptide combinations. 721.174 cells were incubated with the indicated peptides and used as target cells in degranulation assays for CD158b‐positive NK cells from eight KIR2DL3 homozygous donors and six KIR2DL2 homozygous donors. The individual responses of KIR2DL2 (circles) and KIR2DL3 (squares) donor NK cells to (A) VAP‐FA, (B) VAP‐RA, and (C) VAP‐DA are shown. The dashed line connects the mean of the results from the KIR2DL2 homozygous donors and the full line the KIR2DL3 donors. (D–F) Responses to pairs of peptide are shown with the final concentration of peptides in all mixes being 10 μM. The concentration of each of the peptides is indicated on each x‐axis. Data show the mean value for each individual donor, and are from one experiment performed in duplicate. *p* values for the differences between the donors as determined by ANOVA are indicated. For comparisons at individual peptide concentration *0.05 < *p* < 0.1 and **p < 0.001. Degranulation data are normalized to 721.174 with no peptide.

We have used the 721.174 cell line to study the interaction of KIR with group 1 HLA‐C. This cell line expresses HLA‐C*0102, but as it is deficient in TAP, the HLA on the cell surface is expressed with low affinity peptide, and can therefore be exogenously loaded with peptide. Using this cell line for a peptide screen we identified VAPWNSFAL (VAP‐FA) as a high affinity ligand for KIR, VAPWNSDAL (VAP‐DA) as a low affinity ligand and VAPWNSRAL (VAP‐RA) as having an intermediate affinity for both KIR2DL2 and KIR2DL3 (Fig. 1A–C) 23. The peptides have similar affinities for HLA‐Cw*0102 the allele naturally expressed on 721.174 cells (Fig. 1A and Supporting Information Fig 1). This binding correlates well with function in that VAP‐FA is strongly inhibitory, VAP‐RA is weakly inhibitory and VAP‐DA is noninhibitory for both KIR2DL2 and KIR2DL3 homozygous donors (Fig. 1D–F). To investigate whether different donors had different responses to single peptides, 721.174 cells were loaded with VAP‐FA, VAP‐RA, or VAP‐DA and used as targets for CD158b+ NK cells from eight KIR2DL3 homozygous and six KIR2DL2 homozygous donors in degranulation assays. VAP‐FA and VAP‐RA inhibited CD158b‐positive NK cells from both sets of donors at all peptide concentrations (Fig. 2A–C). This is consistent with our previous data showing that in a single peptide model, inhibition is determined by levels of MHC class I expression, and this starts to decline significantly only at levels of less than 2 μM peptide. However, there was a difference in the level of inhibition between the two donor groups for the weakly inhibitory VAP‐RA peptide (*p* = 0.01). This was more evident at higher levels of VAP‐RA in which NK cells from the KIR2DL2‐positive donors were marginally more inhibited than those from KIR2DL3‐positive donors (8 μM VAP‐RA [*p* < 0.001] or 10 μM VAP‐RA [0.1 > *p* > 0.05]) (Fig. 2B). Double peptide experiments were conducted using a final peptide concentration of 10 μM and varying the relative proportions of the two peptides. Consistent with our previous data VAP‐DA behaved as a peptide antagonist in that it reduced inhibition due to VAP‐FA, despite not binding to KIR2DL2‐Fc or KIR2DL3‐Fc, or inhibiting KIR2DL2‐ or KIR2DL3‐positive NK cells. For instance, at 4 μM VAP‐FA alone CD107a expression was approximately 40% of maximum in the absence of VAP‐DA (Fig. 2A), but in its presence (VAP‐DA 6 μM) CD107a expression was 80% (Fig. 2D). Overall CD158b‐positive NK cells from both KIR2DL2‐ and KIR2DL3‐positive donors were inhibited at similar levels for the VAP‐FA/VAP‐DA and VAP‐FA/VAP‐RA ratios tested (Fig. 2D and E). However, we observed small but significantly increased inhibition of NK cells from the KIR2DL2‐positive donors for VAP‐RA/VAP‐DA mixtures overall (*p* < 0.03 (two‐way ANOVA)) (Fig. 2F). Thus, similar to the single peptide experiments significant differences between the donors were seen with the weak inhibitory peptide VAP‐RA, rather than the strongly inhibitory peptide VAP‐FA.

### Differences between KIR2DL2‐ and KIR2DL3‐positive NK cells are greater at lower levels of inhibition

**Figure 3:**
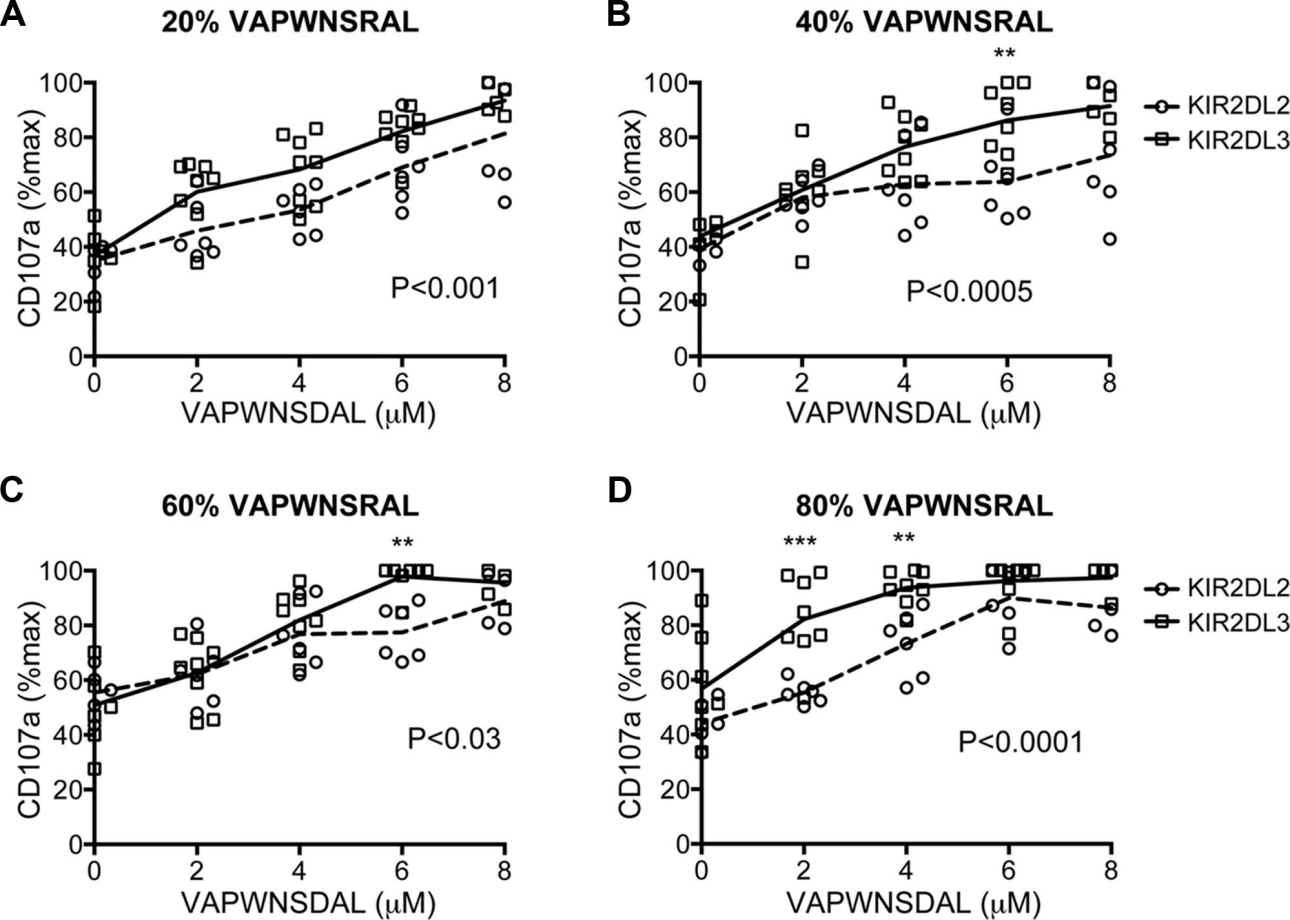
Comparison of inhibition of CD158b+ NK cells from KIR2DL2+ and KIR2DL3+ donors to different combinations of VAP‐FA, VAP‐DA, and VAP‐RA. (A–D) 721.174 cells were incubated with combinations of three peptides as shown in Table 1 and used as target cells in degranulation assays for CD158b‐positive NK cells from KIR2DL2 (circles) and KIR2DL3 (squares) homozygous donors. The mean CD107a expression levels normalised to no peptide are connected by a dashed line (KIR2DL2 homozygous donors) or a full line (KIR2DL3 homozygous donors). The ratios of VAP‐RA to VAP‐FA were: (A) 20% VAP‐RA:80% VAP‐FA, (B) 40% VAP‐RA:60% VAP‐FA, (C) 60% VAP‐RA:40% VAP‐FA, and (D) 80% VAP‐RA:20% VAP‐FA. In all experiments, the VAP‐RA:VAP‐FA ratio was kept constant but the total quantity was varied in combination with VAP‐DA in order to maintain a total peptide concentration of 10 μM in the peptide mix (Supporting Information Table 1). Data show the mean value for each individual donor, and are from one experiment performed in duplicate. *p* values for the differences between the donors as determined by ANOVA are shown. For comparisons at individual peptide concentrations *p < 0.05, **p < 0.01, ***p < 0.001.

In order to explore the effects of changing class I‐bound peptide in more detail we devised experiments using peptides of all three categories: strongly inhibitory, weakly inhibitory, and antagonistic. These experiments were conducted using fixed ratios of weak/strong inhibitory peptides (VAP‐RA:VAP‐FA) and then gradually increasing the amount of antagonistic VAP‐DA in the mix (Supporting Information Table 1). All experiments were performed at a final peptide concentration of 10 μM. Overall there was a highly significant difference between the two groups of donors as determined by two‐way ANOVA (*p* < 0.0001) (Fig. 3). For each ratio of VAP‐RA:VAP‐FA overall there were statistically significant differences in the change in inhibition of CD158b‐positive NK cells induced by the addition of VAP‐DA between the KIR2DL2 and KIR2DL3 homozygotes (Fig. 3). However, the patterns varied with VAP‐RA:VAP‐FA ratio. When the strongly inhibitory peptide predominated (20%:80% VAP‐RA:VAP‐FA) there were small, but individually insignificant differences induced by the antagonist between the donor groups at all VAP‐RA:VAP‐FA ratios (Fig. 3A). For 40%:60% and 60%:40% VAP‐RA:VAP‐FA the differences were more evident at higher concentrations of VAP‐DA, and reached statistical significance at 6 μM VAP‐DA (*p* < 0.05 and *p* < 0.01, respectively) (Fig. 3B and C). When the weakly inhibitory peptide predominated (VAP‐RA:VAP‐FA, 80%:20%), differences induced by the antagonist were more evident at low concentrations of VAP‐DA and were significant at both 2 μM and 4 μM (*p* < 0.001 and *p* < 0.01, respectively) (Fig. 3D). Degranulation saturates at higher concentrations of VAP‐DA therefore differences are no longer apparent. Thus, in general, differences between the donors were observed at lower levels of inhibition. Analyzing the subgroup of donors with ligands for KIR2DL2 and KIR2DL3 (group 1 HLA‐C positive) gave similar results to the overall cohort (Supporting Information Fig. 2).

### Response to changes in peptide repertoires discriminates KIR2DL2 from KIR2DL3‐positive donors

In order to describe these data quantitatively, we constructed a regression model. The model assumes that the degranulation (A), quantified by CD107a in a population of NK cells, varies with concentrations of FA, DA, and RA peptides as,

(1) A=100%−kF FA −kD DA −kR RA +k DF  DA  FA +k FR  FA  RA +k RD  RA  DA +k DFR  DA  FA  RA .

First we evaluated the parameters in the model in the absence of any cubic order term, i.e. k_DFR_ = 0. We also set k_DA_ = 0 as DA minimally affects activation when NK cells are stimulated with DA peptide alone. When searching for parameter values we restricted the search to only positive values as the peptides have been shown to induce inhibition in single peptide experiments (k_F_, k_D_, k_R_) and antagonize inhibition in two and three peptide experiments (k_DF_, k_FR_, k_RD_, k_DFR_). The model in Eq. (1) agreed slightly better with the data from KIR2DL3 donors compared with the KIR2DL2 donors. This is reflected in the larger values of χ^2^ (Table 1) produced for the KIR2DL2 data.

**Table 1:** . Parameter values obtained by minimizing χ^2^

Parameter values	KIR2DL2 donor	KIR2DL3 donor
k_F_	6.4	6.05
k_D_	0	0
k_R_	5.27	2.36
k_DF_	0.003	0.0007
k_FR_	0.018	0.0016
k_RD_	0.125	0.54
k_DFR_	0	0
χ^2^	12.6	8

From the parameter values in Table 1, the [FA]–[DA] and [FA]–[RA] pairs appear to have less impact in inducing activation compared with the [DA]–[RA] pair in the triple peptide experiments. The qualitative features of the model did not change when we added the cubic term in estimating the model parameters. Thus the [RA]–[DA] pair seemed to exert more influence in activation compared with the other two pairs.

**Figure 4:**
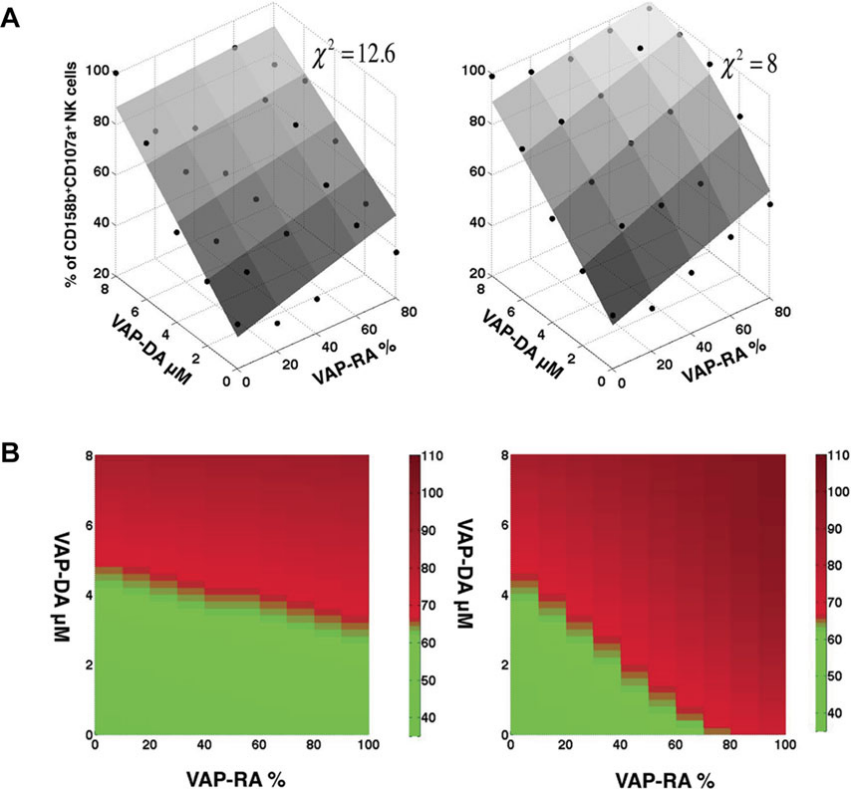
Regression modeling of experimental data from assays of inhibition using the triple mix peptide combinations. (A) Comparison between the model and the experiments. The activation determined by the model (gray surface) in Eq. (1) for the parameter values noted in Table 1 when the cubic term in Eq. (1) was set to zero. The points show the measured %CD107a degranulation averaged over multiple trials (*n* ≥ 3). The left panel shows the comparison for KIR2DL2‐positive donors and the right panel for KIR2DL3‐positive donors. (B) Comparison of the regression models for KIR2DL2 and KIR2DL3‐positive donors. The peptide combinations determined by Eq. (1) that produce greater than 65% degranulation as compared with no peptide for KIR2DL2 donors (left panel) and KIR2DL3 donors (right panel). Degranulation of more than 65% is shown in red, and less than 65% is shown in green. Computer generated graphics from data in Fig. 3 are shown.

The fitted function shows that KIR2DL3 expressing cells are activated more easily than the KIR2DL2 expressing cells (Fig. 4A). This is shown in Fig. 4B in which the peptide combinations that produce more than 65% CD107a degranulation are shown in red. This shows that activation stays below 65% (shown in green) for a greater fraction of peptide combinations for KIR2DL2‐positive NK cells, compared with KIR2DL3‐positive NK cells. Thus 12 out of 25 peptide combinations for KIR2DL2 donors and 7 out of 25 peptide combinations for KIR2DL3 donors yielded a degranulation value of less than 65%. The role of the [DA]‐[RA] pair seems to be greater for KIR2DL3 (K_RD_ = 0.54) compared with KIR2DL2 (K_RD_ = 0.125). This is also consistent with the single peptide data in which K_R_ for KIR2DL2 is approximately twice that for KIR2DL3. Based on the data in Table 1 the differences in inhibition between KIR2DL3 and KIR2DL2 donors can be given by:

(2) Inhibition 2DL2−2DL3=−2.91 RA −0.415 RA  DA −0.35 FA −0.004 DA  FA +0.00164 FA  RA

Therefore the [RA] term is dominant over the [FA] term and the [RA][DA] term dominant over [FA][DA]. Thus these data imply that KIR2DL3‐positive NK cells respond more readily to changes in peptide repertoire than KIR2DL2‐positive NK cells, and the difference in inhibition between donors is determined by the concentrations of the weak agonist peptide and the weak agonist/antagonist peptide combination.

## Discussion

We have used a model system to investigate how NK cells may respond to changes in peptide repertoire. We have assumed that there are four broad classes of peptide/MHC class I with respect to KIR: strong inhibitory, weak inhibitory, antagonistic, and null, with null peptides not contributing to NK cell signaling. We, therefore, modeled changes in peptide repertoire using representatives of the three classes of peptide that could modulate changes in NK cell activity. It has previously been shown that CD107a degranulation correlates well with binding using KIR‐Fc fusion constructs and that the selected peptides are representative of these groups, with VAP‐FA being the strongest binder in our peptide screen, VAP‐DA being one of the nonbinders and VAP‐RA being intermediate between the two 23. Our experimental and modeling data indicate that there are differences in the way that CD158b‐positive NK cells from donors with different KIR genotypes respond to changes in peptide repertoire.

It has been shown previously that KIR2DL2 has a greater affinity for HLA class I than KIR2DL3 due to a difference in the hinge angle between the two domains of the receptors 18, 19. However, functional experiments did not detect differences in inhibition by group 1 HLA‐C alleles between KIR2DL2 and KIR2DL3‐positive NK cells. These experiments were performed using transfectant cell lines expressing single HLA‐C alleles, which are strongly inhibitory, and this is consistent with our observations that VAP‐FA is not discriminatory between the two donor genotypes. Our data show that functional differences are more subtle and related to the peptides presented by MHC class I. Thus, in single peptide experiments both sets of donors made similar responses to VAP‐DA and VAP‐FA, but not to VAP‐RA. In the triple mix experiments, the ratio of VAP‐RA to VAP‐DA appeared to be the most discriminatory between the donors, but in the mathematical analysis the concentration of VAP‐RA appeared dominant over the combination of the two.

The observed differences between the donors are small. However, given that KIR2DL2 and KIR2DL3 differ on average by only 4 amino acids in the D1 and D2 domains this is not surprising. Additionally, large immunogenetic studies have been required to define the protective effects of KIR against infection, arguing against individual KIR having dramatic effects and demonstrating the subtly of functional polymorphisms within the KIR system.

Our data show that in this model system changes in peptide repertoire lead more readily to activation of KIR2DL3‐positve NK cells than KIR2DL2‐positive NK cells. Testing of these data with additional peptide/HLA combinations would assist in establishing the broad applicability of these findings. Furthermore, the in vivo significance of this work needs further exploration. However, changes in peptide repertoire may occur during viral infection or tumorigenesis through the presentation of viral antigens, tumor antigens or self‐peptides 26. The formation of the MHC class I peptide repertoire is complex and appears to depend on a combination of the degradation of mature proteins, and also recently synthesized misfolded proteins (defective ribosomal products “DRiPs”) 27, 28. Viral infection can readily change the MHC class I peptide repertoire either by presentation of viral peptides, but also by changing the self‐peptides presented by MHC class I. Moreover, the induction of the immunoproteosome by proinflammatory signals during an active immune response is likely to significantly alter the peptide landscape presented by MHC when compared with noninflammatory conditions. At an early time point during vaccinia infection, nearly 50% of the peptide repertoire of MHC class I is composed of viral peptides 29. Therefore, changes in peptide repertoire during viral infection can be substantial and, based on our data, potentially sufficient to activate an NK cell. Peptide antagonism is a mechanism that permits detection of changes in peptide repertoire and this would be more readily accomplished in the presence of weaker inhibitory peptides. Based on our peptide‐binding screen, the subset constituting the inhibitory peptides form a continuum of which VAP‐RA is at the weaker KIR‐binding and VAP‐FA at the stronger KIR‐binding ends. Thus, although other peptides may have subtly different effects the peptide combinations tested broadly represent changes in MHC class I peptide repertoire.

The modeling data provide a template for how the inhibition of NK cells may change in response to the peptide content of MHC class I and the better fit of the model in Eq. (1) with the data for KIR2DL3 compared with KIR2DL2, could indicate the presence of additional factors, that modulate NK cell function for only KIR2DL2 donors. One such factor could be KIR2DS2, which is also bound by the CD158b antibody used in this study. KIR2DS2 is in strong linkage disequilibrium with KIR2DL2, therefore all our KIR2DL2 donors also had this activating receptor. It has recently been shown that HLA‐A11 is a ligand for KIR2DS2 and that KIR2DS2‐positive NK cell clones can recognize HLA‐Cw*0304 30, 31. However, we were unable to confirm or refute a role for KIR2DS2 as excluding these individuals meant that numbers were too small for statistical analysis. However, the affinity of KIR2DS2 for HLA class I is much lower than for KIR2DL2 22 and in our experiments NK cells from the KIR2DL2 donors were inhibited to a greater extent than those from the KIR2DL3 donors. Taken together, this suggests that the effect of KIR2DS2 in these experiments is likely to be small, if any.

KIR2DL3, but not KIR2DL2 has been shown to be protective against viral infection such as hepatitis C virus and also against fulminant malaria. It is thought that the selection for the KIR A haplotype, containing KIR2DL3, is related to defense against pathogens. Our data suggest that fine tuning of NK cell activity by MHC class I peptide may discriminate KIR2DL3, from KIR2DL2, positive donors in a manner that allows marginally greater reactivity of KIR2DL3‐positive NK cells. These subtle differences may be important for understanding how KIR contribute to the genetic susceptibility to infectious disease.

## Materials and methods

### Peptide stabilization assay

The HLA‐Cw*0102‐positive, TAP deficient 721.174 cell line was cultured in RPMI medium 1640 supplemented with 1% penicillin/streptomycin (Invitrogen, Paisley, UK) and 10% fetal calf serum (Lonza). For peptide stabilization, 2 × 10^5^ 721.174 cells were incubated with or without synthetic peptides (GL Biochem, Shanghai Ltd., China) overnight at 26°C. After 16 h, cells were washed and stained with a primary HLA‐Cw*0102‐specific antibody VP6G3 for 1 h at room temperature, followed by a secondary FITC‐conjugated goat anti‐human IgM antibody (AbD Serotec) for 30 min at room temperature. Cells were then washed and fixed in 1% PFA/ fetal calf serum. Cells were analyzed by flow cytometry (BD Biosciences, Oxford, UK). The mean intensity of fluorescence of 10 000 events was collected.

### KIR2DL2/3‐Fc binding assay

KIR2D2L2‐IgG and KIR2DL3‐IgG fusion constructs (KIR2DL2‐Fc & KIR2DL3‐Fc Chimera; R&D Systems) were conjugated with protein A Alexa Fluor 488 (Invitrogen) at a molar ratio of 6:1. A total of 1 × 10^6^ 721.174 cells were incubated with 10 μM peptide as described above and then stained with KIR‐Fc. After fixation in 4% w/v paraformaldehyde cells were analyzed by flow cytometry.

### CD107a NK cell degranulation assay

Human PBMC were isolated from the blood of 14 healthy donors using Hypaque‐Ficoll (GE Healthcare, Amersham, UK) density centrifugation, with informed consent and full ethical approval (REC ref 06/Q1701/120). A total of 3 × 10^5^ PBMCs were stimulated overnight with 1 ng/mL recombinant human IL‐15. Peptide pulsed 721.174 targets were prepared as for the stabilization assays. Target cells were resuspended with PBMCs at an effector‐to‐target (E:T) ratio of 5:1 in fresh R10 medium containing peptide and anti‐CD107a‐Alexa‐fluor‐647 antibody (eBioscience, Hatfield, UK). Cells were incubated for 1 h at 26°C, then 6 μg/mL Golgi‐Stop^TM^ (BD Biosciences) was added, and incubated for a further 3 h at 26°C. Cells were washed in wash buffer (1% BSA/0.1% NaN_3_) and blocked with blocking buffer (10% human serum in PBS) for 30 min and then stained with the following antibodies: anti‐CD3‐PerCP (Biolegend, San Diego, USA), anti‐human CD56‐PE, and anti‐human CD158b‐FITC (both BD Biosciences). Cells were fixed in 1% PFA and analyzed by flow cytometry. Individual assays for each donor were performed once in duplicate and the mean value used for subsequent analysis.

### KIR and HLA genotyping

All donors were typed for KIR using a quantitative PCR comparative Ct method 32. Donors were typed for HLA‐B and HLA‐C at the Anthony Nolan Institute (London, UK) using Luminex xMAP**®** technology.

### Statistics

These were calculated using GraphPad Prism by two‐way ANOVA to compare KIR2DL2 and KIR2DL3 homozygous donors, with a post Student's *t*‐test and Bonferroni correction to compare individual data points.

### Data modeling

We constructed a regression model using the experimental data. The model assumes that the degranulation (A), quantified by CD107a in a population of NK cells, varies with concentrations of FA, DA, and RA peptides as:

(1) A=100%−kF FA −kD DA −kR RA +kDF DA  FA +kFR FA  RA +kRD RA  DA +kDFR DA  FA  RA

In the above expression, [x] denotes the concentration of a peptide, x; k_F_, k_D_, k_R_, k_DF_, k_FR_, k_RD_, and k_DFR_ are model parameters that are evaluated by fitting the above expression to the data characterizing NK cell activation stimulated by mixtures of the three peptides. The terms linear in the concentrations in Eq. (1) describe inhibition of NK cell activation when NK cells are stimulated individually by the peptides. The terms proportional to the product of two peptide concentrations describe the antagonistic effect induced by the peptides. The last term describes the effect of a triple peptide stimulation that cannot be captured by pair‐wise stimulations of the peptides. NK cell activation is assumed to be 100% in the absence of any DA, FA or RA peptide. We determine the parameters in Eq. (1) by fitting the expression with the data from the three‐peptide mixture experiments. Then we use Eq. (1) to address two principal issues:

(1) To quantify differences between the data generated from the KIR2DL2 and the KIR2DL3 donors in terms of the parameter values.

(2) To quantify differences between the ability of the NK cells from the KIR2DL2 and the KIR2DL3 donors to respond when the peptide mixtures stimulate the cells.

We calculated the parameters in Eq. (1) by minimizing χ^2^ as defined below. χ^2^ quantifies the difference between the model and the experimental data.

(3) χ2=∑iAi expt −Ai model σi expt 2

where, *i* refers to an experiment with any mixture of the three peptides, FA, DA, and RA. A*_i_* ^expt^ denotes the average value of the % of CD107a degranulation measured over multiple trials (*n* > 3) for a particular mixture of peptides indexed by *i*. σ*_i_* ^expt^ denotes the variance in the measured % of CD107a degranulation in the trials done in the *i* ^th^ experiment. In the experiments, the peptide combinations were chosen such that the total concentration of the peptides was held fixed at 10 μM, i.e. [FA]+[DA]+[RA] = 10 μM. A*_i_* ^model^ represents the % of CD107 degranulation calculated from Eq. (1) for the peptide concentrations corresponding to the *i* ^th^ experiment. We chose the parameters that minimized χ^2^ in Eq. (3). The minimization was carried out by the routine *lsqcurvefit* in MATLAB.

**Abbreviations**
KIR: killer cell immunoglobulin‐like receptors
VAP‐FA: VAPWNSFAL
VAP‐RA: VAPWNSRAL
VAP‐DA: VAPWNSDAL

## Supporting information

As a service to our authors and readers, this journal provides supporting information supplied by the authors. Such materials are peer reviewed and may be re‐organized for online delivery, but are not copy‐edited or typeset. Technical support issues arising from supporting information (other than missing files) should be addressed to the authors.

Figure 1.

Peer review correspondence
