# Supplementary material for: Peptide selectivity discriminates NK cells from KIR2DL2‐ and KIR2DL3‐positive individuals
Source: Eur J Immunol. 2014 Nov 29;45(2):492–500. doi: 10.1002/eji.201444613 (PMC4324016; doi:10.1002/eji.201444613)
Supplement: Supplementary file 1 — Figure 1. [file EJI-45-492-s001.pdf]

European Journal of Immunology

Supporting Information
for
DOI 10.1002/eji.201444613

Sorcha Cassidy, Sayak Mukherjee, Thet Mon Myint, Berenice Mbiribindi, Helen North, James Traherne, Arend Mulder, Frans HJ Claas, Marco A Purbhoo, Jayajit Das and Salim I Khakoo

Peptide selectivity discriminates NK cells from KIR2DL2- and KIR2DL3-positive individuals

© 2014 The Authors. *European Journal of Immunology* published by WILEY-VCH Verlag GmbH & Co. KGaA, Weinheim

Supporting Information Table 1: *Peptide concentrations used in the triple mix experiments*

DA (µM)	RA(µM)	FA(µM)	RA:FA (%)
0	2	8	20:80
0	4	6	40:60
0	6	4	60:40
0	8	2	80:20
2	1.6	6.4	20:80
2	3.2	4.8	40:60
2	4.8	3.2	60:40
2	6.4	1.6	80:20
4	1.2	4.8	20:80
4	2.4	3.6	40:60
4	3.6	2.4	60:40
4	4.8	1.2	80:20
6	0.8	3.2	20:80
6	1.6	2.4	40:60
6	2.4	1.6	60:40
6	3.2	0.8	80:20
8	0.4	1.6	20:80
8	0.8	1.2	40:60
8	1.2	0.8	60:40
8	1.6	0.4	80:20

Supporting Information Figure 1

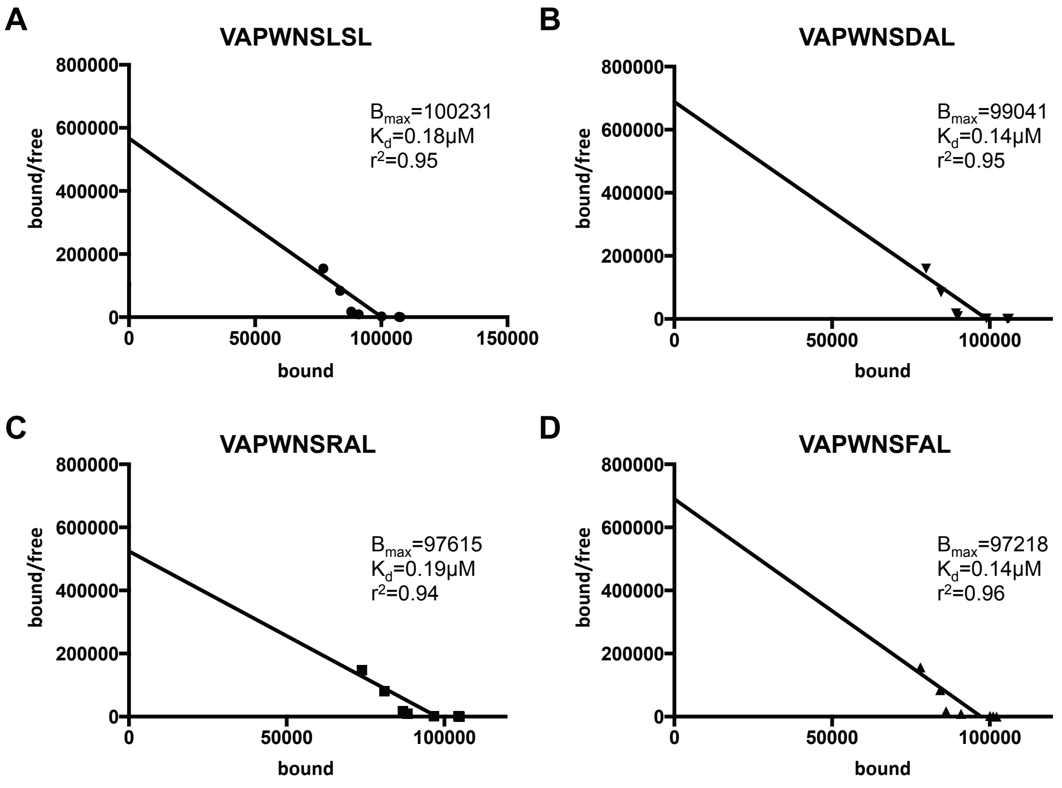

*Affinities of VAPWNSLSL derivative peptides for HLA-C*0102*

Scatchard plots derived from stabilisation experiments of HLA-Cw*0102 and analysis by flow cytometry using V6PG3. Panels A-D show the Scatchard plots for the data in Figure 1A and the associated regression line given by $Y = Bmax*X/(K_d + X)$[GraphPad Prism6®] for the individual peptides. The dissociation constant (K_d), maximal binding (B_{max}) and correlation coefficients (r^2) of the regression lines are shown.

Supporting Information Figure 2

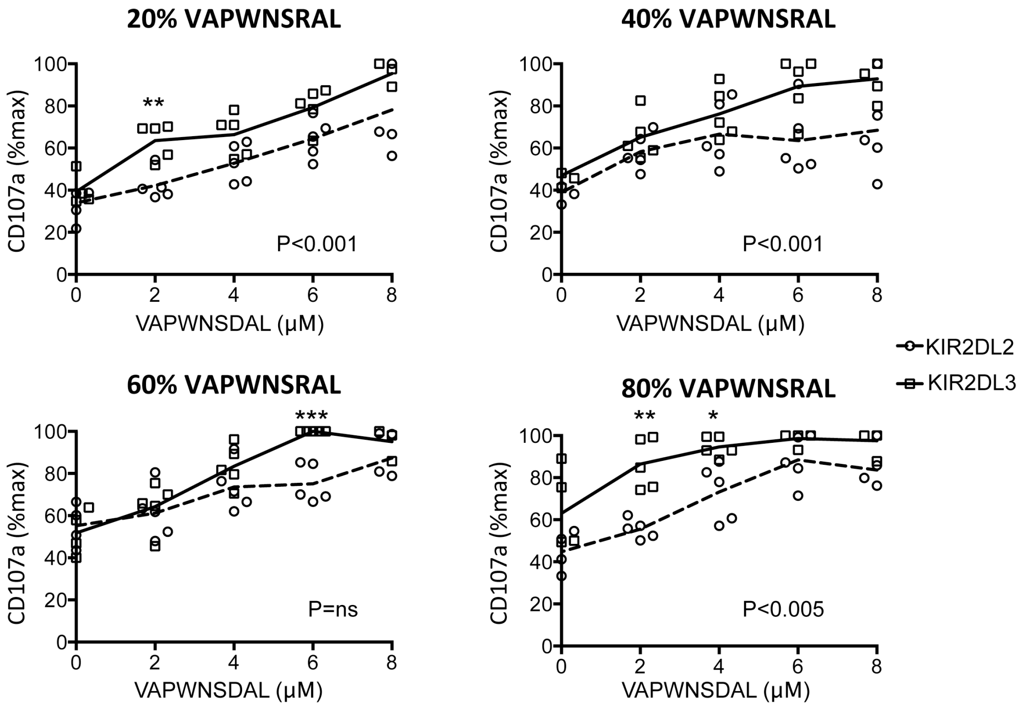

*Responses to changes in peptide repertoire for group 1 HLA-C-positive HLA-A*11-negative donors.*

Data from Figure 3 for the five KIR2DL2 (circles) and five KIR2DL3 (squares) homozygous donors with ligands for KIR2DL2/3 but not KIR2DS2. The mean CD107a expression normalised to no peptide is shown by a dashed line (KIR2DL2 homozygous donors) or a full line (KIR2DL3 homozygous). Peptide concentrations are as for Figure 3 and indicated in Table 1. P values for the differences between the donors as determined by ANOVA are shown. For comparisons at individual peptide concentrations *p<0.05, **p<0.01, ***p<0.001.
