# Supplementary material for: Peptide selectivity discriminates NK cells from KIR2DL2‐ and KIR2DL3‐positive individuals
Source: Eur J Immunol. 2014 Nov 29;45(2):492–500. doi: 10.1002/eji.201444613 (PMC4324016; doi:10.1002/eji.201444613)
Supplement: Supplementary file 2 — Peer review correspondence [file EJI-45-492-s002.pdf]

European Journal of Immunology

Peer review correspondence

Peptide selectivity discriminates NK cells from KIR2DL2- and KIR2DL3-positive individuals

Sorcha Cassidy, Sayak Mukherjee, Thet Mon Myint, Berenice Mbribindi, Helen North, James Traherne, Arend Mulder, Frans HJ Claas, Marco A Purbhoo, Jayajit Das and Salim I Khakoo

Correspondence: Prof. Salim Khakoo, Faculty of Medicine, University of Southampton, Mailpoint 811, Level E South Academic Block, Southampton General Hospital, Tremona Road, Southampton, SO16 6YD, UK

Review Timeline:	Submission date:	3 March 2014
	First Editorial decision:	4 April 2014
	First Revision received:	19 September 2014
	Accepted:	24 October 2014

Handling Executive Committee member: Prof. Marco Colonna

Please note that the correspondence below does not include the standard editorial instructions regarding preparation and submission of revised manuscripts, only the scientific revisions requested and addressed.

First Editorial Decision – 4 April 2014

Dear Dr. Khakoo,

Manuscript ID eji.201444613 entitled "Peptide selectivity discriminates NK cells from KIR2DL2- and KIR2DL3-positive individuals" which you submitted to the European Journal of Immunology has been reviewed. The comments of the referees are included at the bottom of this letter.

A revised version of your manuscript that takes into account the comments of the referees will be reconsidered for publication.

You should also pay close attention to the editorial comments included below. In particular, please edit your figure legends to follow Journal standards as outlined in the editorial comments. Failure to do this will result in delays in the re-review process.

Please note that submitting a revision of your manuscript does not guarantee eventual acceptance, and that your revision will be re-reviewed by the referee(s) before a decision is rendered.

European Journal of Immunology

If the revision of the paper is expected to take more than three months, please inform the editorial office. Revisions taking longer than six months may be assessed by new referee(s) to ensure the relevance and timeliness of the data.

Once again, thank you for submitting your manuscript to European Journal of Immunology and we look forward to receiving your revision.

Yours sincerely,
Laura Soto Vazquez

On behalf of Prof. Marco Colonna

Editorial Office
European Journal of Immunology
e-mail: ejied@wiley.com
www.eji-journal.eu
****
Reviewer: 1

Comments to the Author
Cassidy et al. demonstrate effects of peptides on the function of KIR2DL-expressing NK cells. They clearly show that peptides bound to HLA-Cw*0102 affect KIR2DL recognition but they have already published similar data. New findings on this paper are the analyses of NK cells from seven KIR2DL2 or six KIR2DL3 homozygous donors. Although the authors conclude that there are significant differences between NK cells from KIR2DL2 or KIR2DL3 homozygous donors, the differences are so small. Especially, significant differences are observed only when certain concentrations of peptides were used. In addition, it is not described how many times they have repeated the experiments. Therefore, it is unclear whether the differences they found are physiologically relevant.

The authors described that they used NK cells from seven 2DL2 or six 2DL3 homozygous donors. It is unclear whether they used pooled NK cells from several donors or they analyzed NK cells from individual donors and presented the average. Because the differences between 2DL2 and 2DL3 are so small, they should show data of NK cells from individual 13 donors in Fig. 2 and 3, and calculate statistical significances. Otherwise, it cannot be concluded that the differences are really significant.

In Fig. 4A, the ranges of proportions of CD107a positive cells (Z-axis) are different between 2DL2 and 2DL3. The ranges should be the same between them. It looks like that they modify a small difference to big.

In Fig. 4B, they show that there is a significant difference between 2DL2 and 2DL3 using a regression model. However, the difference mainly comes from the differences of 2DL2 and 2DL3 in Fig. 3D. Therefore, the authors should show more rigid data in Fig.2 and Fig.3 as described above.

Minor:
In introduction, they cite reference 25 but it is not related.

Reviewer: 2

Comments to the Author
Cassidy et al. provide some elegant data and mathematical modeling analysis to demonstrate that the lower affinity KIR2DL3 is more sensitive than KIR2DL2 to variations in the inhibitory or antagonistic nature of peptides loaded in HLA-C. Although some of the differences are subtle, the data are very tight and some statistically significant differences were noted that are of biological interest. The data and interpretations are mainly straightforward, although a few issues could be addressed to improve the manuscript.

1. Details of the genotypes of donors are vague. Did the 13 donors express HLA-C1 ligands for the KIR2DL2/3 being tested? If not, did lack of ligand or ligand homozygous/heterozygous status alter the responses? Also, given recent findings in the literature, the impact of KIR2DS2 might differ in donors expressing or lacking ligand.

2. At the beginning of the results, the characteristics of the 721.174 cell line (TAP-deficient) should be better explained to orient the reader as to how this cell line permits the peptide loading of HLA-C.

3. Equation (2) appears to be duplicated.

4. The histograms in figure 1 could be reduced in size, whereas the plots should be enlarged to increase resolution.

Reviewer: 3

Comments to the Author

The paper by Cassidy et al argues that there are differences in peptide selectivity between KIR2DL2 and KIR2DL3 with NK cells expressing the latter being more responsive to changes in peptide repertoire than those expressing 2DL2. While the data are broadly consistent with this hypothesis, the experimental system used to show this is extremely artificial and quite limited. Indeed, the paper relies entirely on responses to 3 closely related peptides, which may or may not be representative of different classes of endogenous peptides.

The work itself looks to be technically quite tight – the assays are internally consistent and replicates are very close. Indeed the data appear to assign statistical significance to data that in magnitude looks to be barely different (eg Figs 2B, F)

Fig 3 extends this analysis to more complex mixtures (3 peptides rather than 2) but the result is a slight broadening of the differences in response between 2Dl2+ and 2DL3+ NK cells, the data being broadly consistent with the idea that 2DL2 interacts with C1 with higher affinity than 2DL3 but nevertheless the differences are still modest with perhaps the 80% mix of VAPWNSRAL being the one exception where the differences are clearly appreciable. Moreover the authors provide an argument for why this context might be one in which the impact of VAPMWNSDAL on inhibition is more marked.

It's not clear the mathematical modeling reveals anything that the primary data themselves do not.

I would think the analysis needs broadening to include a number of additional peptides to be comfortable that the conclusions are sound.

Reviewer: 4

Comments to the Author

Peptide selectivity discriminates NK cells from KIR2DL2- and KIR2DL3-positive individuals.

In a continuation of their previous work (Fadda et al. PNAS 2010), the manuscript by Cassidy et al describes the results of a peptide competition assay in the context of NK cell degranulation. The authors have continued to use a model system comprised of three peptides and have extrapolated those findings to make general comments on the role of peptide repertoire on the activation of KIR2DL2 vs KIR2DL3 positive NK cells. The major concern regarding this paper is the level of conceptual advance made in

European Journal of Immunology

comparison to their previous work (Fadda et al., PNAS 2010) – indeed, the current paper rests heavily on this prior work.

To exemplify, the 2010 PNAS paper states in the discussion:
"KIR2DL3-positive NK cells may therefore be sensitive to a wider spectrum of changes in peptide repertoire than KIR2DL2-positive NK cells as a greater fraction of peptides could be antagonistic. This may in part explain the genetic association of KIR2DL3, but not KIR2DL2, with protection from hepatitis C infection"

While the abstract of this manuscript states: "KIR2DL3 positive NK cells were more sensitive to changes in the peptide content of MHC class I than KIR2DL2-positive NK cells."

A major discriminating factor between the two pieces of work appears to be the mathematical modeling applied to this paper, and this may not be considered sufficiently suitable to warrant publication in EJI.

Some general points:

1) It is not clear whether the DA peptide is a low affinity antagonistic peptide or a no-affinity peptide. Further to this, there appear to be differences in affinity for the RA peptide between KIR2DL2 and KIR2DL3 (Figure 1). This could go someway to explaining differences observed between these KIR2DLs. Precise details about the binding affinity would strengthen the paper.

2) A confounding variable that has not been addressed is the allele of KIR2DL that is carried by the donors. Previous studies have observed that KIR2DL polymorphism can significantly impact on HLA-C recognition and signalling. What is presented in this study is a mean and SEM for a number of donors. It would be interesting to see the distribution by plotting all donors. Further, if the alleles of the donors are known then they should be presented.

3) Figure 1A provides evidence that all the peptides bind HLA-C. However, can the authors comment on the relative affinity of the peptides for HLA-C? If the DA peptide has a higher affinity than the other peptides then there will be more on the cell surface than the ratios added would suggest.

4) Discussion: Paragraph 3:
The line " The implications of these data are that changes in peptide repertoire lead more readily to activation of KIR2DL3… than KIR2DL2 positive NK cells". This statement is perhaps too strong and should be limited to the model system that was used. Especially when considered in the absence of affinity data, the absence of the alleles of KIR2D being presented and the limitations of the study to just three peptides.

European Journal of Immunology

First Revision – authors' response – 26 August 2014

Point by point reply to reviewers

We thank the reviewers for their comments and have underlined the changes we have made to the manuscript. We have amended Figures 1-4 as requested, and included a new supplementary figure to show an analysis of peptide binding to HLA-C*01. We attach our point-by point reply below.

Reviewer: 1

Comments to the Author
Cassidy et al. demonstrate effects of peptides on the function of KIR2DL-expressing NK cells. They clearly show that peptides bound to HLA-Cw*0102 affect KIR2DL recognition but they have already published similar data. New findings on this paper are the analyses of NK cells from seven KIR2DL2 or six KIR2DL3 homozygous donors. Although the authors conclude that there are significant differences between NK cells from KIR2DL2 or KIR2DL3 homozygous donors, the differences are so small. Especially, significant differences are observed only when certain concentrations of peptides were used. In addition, it is not described how many times they have repeated the experiments.

We agree that with this reviewer that the differences are small, but as noted by Reviewer 2 "the data are very tight and some statistically significant differences were noted that are of biological interest".
To achieve this we performed each assay once from each donor in duplicate and calculated the mean CD107a expression as a percentage of the maximum. We then calculated the mean and standard errors of the mean from each donor. We have been careful in our statistical analysis to perform an ANOVA test (to compare the overall findings for each peptide or peptide combination) and then used students T-test with correction for multiple comparisons between the donors in order to determine the differences for individual peptide concentrations. We have detailed how the assay was performed in the methods section (P25, L21). "Individual assays for each donor were performed once in duplicate…" Please note also that we have studied fourteen donors (six 2DL2 and eight 2DL3 homozygotes), not thirteen as we originally wrote in the manuscript. We have therefore amended the manuscript at the relevant places to reflect this error.

Therefore, it is unclear whether the differences they found are physiologically relevant.

This is a very reasonable point as it is unclear exactly what physiologically relevant means in terms of the KIR system. The starting point for our work was that in a number of important infectious diseases (eg HCV, malaria and chikungunya virus) KIR2DL2 and KIR2DL3 confer different outcomes, and whilst it is clear that these receptors have different affinities for the group 1 HLA-C allotypes in binding assays,

European Journal of Immunology

differences in inhibition have not been noted using previously described experimental systems, mainly 721.221 single allele transfectants. We therefore approached this using our peptide mix experiments and found small statistically significant differences between donors with different genotypes. However to my knowledge it is not clear as to the extent to which peptide repertoire in the context of HLA-C changes during these infections and we agree that the physiological relevance is difficult to ascertain. However we still feel it is important to note differences in function between KIR2DL2 and KIR2DL3-positive donors due to the associations with disease outcome. Therefore we have added a new paragraph in the discussion "The observed differences between the donors are small…" and go on to explicitly state "Furthermore the in vivo significance of this work needs further exploration." (P21 second paragraph)

The authors described that they used NK cells from seven 2DL2 or six 2DL3 homozygous donors. It is unclear whether they used pooled NK cells from several donors or they analyzed NK cells from individual donors and presented the average. Because the differences between 2DL2 and 2DL3 are so small, they should show data of NK cells from individual 13 donors in Fig. 2 and 3, and calculate statistical significances. Otherwise, it cannot be concluded that the differences are really significant.

We did not use pooled NK cells and have amended Figs 2 and 3 to show individual donor data (means of experiments performed in duplicate).

In Fig. 4A, the ranges of proportions of CD107a positive cells (Z-axis) are different between 2DL2 and 2DL3. The ranges should be the same between them. It looks like that they modify a small difference to big.

We apologize for this oversight and have amended this figure.

In Fig. 4B, they show that there is a significant difference between 2DL2 and 2DL3 using a regression model. However, the difference mainly comes from the differences of 2DL2 and 2DL3 in Fig. 3D. Therefore, the authors should show more rigid data in Fig.2 and Fig.3 as described above.

We have performed this as described above

Minor:
In introduction, they cite reference 25 but it is not related.

Ref 25 describes the elution of VAPWNSLSL, the peptide that formed the basis of our original peptide screen, from HLA-C*0102. We have left it in as we feel it may be helpful to other researchers to be able to refer back to this original data.

European Journal of Immunology

Reviewer: 2

Comments to the Author

Cassidy et al. provide some elegant data and mathematical modeling analysis to demonstrate that the lower affinity KIR2DL3 is more sensitive than KIR2DL2 to variations in the inhibitory or antagonistic nature of peptides loaded in HLA-C. Although some of the differences are subtle, the data are very tight and some statistically significant differences were noted that are of biological interest. The data and interpretations are mainly straightforward, although a few issues could be addressed to improve the manuscript.

1. Details of the genotypes of donors are vague. Did the 13 donors express HLA-C1 ligands for the KIR2DL2/3 being tested? If not, did lack of ligand or ligand homozygous/heterozygous status alter the responses? Also, given recent findings in the literature, the impact of KIR2DS2 might differ in donors expressing or lacking ligand.

We agree that the presence or absence of HLA class I ligands for the KIR may affect reactivity. However, the numbers included in the study are too small for detailed HLA analysis ie stratification by group HLA-C1 ligand homo/heterozygosity. Therefore we have reanalyzed the data for those in whom we have confirmed the presence of an HLA-C ligand, in order to have a relatively homogenous cohort with respect to HLA type (five KIR2DL2 and five KIR2DL3 positive donors). Overall this does not substantially alter our findings ie significant differences remain between KIR2DL2 and KIR2DL3-positive donors for the triple mix of peptides with VAP-RA at 20, 40, and 80μM. However the least significant finding in our previous analysis (VAP-RA 60 μM) is no longer significant. Additionally similar individual data points remain significant in this analysis. We have detailed this in the text (P17, L39) and appended these data as a supplementary figure (Supporting information Fig 2). In terms of KIR2DS2 there are two published ligands that we are aware of: the HLA-A11:KIR2DS2 crystal structure (Liu et al PNAS 2014. 111: 2662-2667) and HLA-C*0304 recognition by KIR2DS2-positive NK cell clones (David et al J Immunol 2013. 191: 4778-4788). None of our donors were HLA-A11 positive but exclusion of the HLA-C*03-positive donors left only 3 individuals per group which was too small for a meaningful statistical analysis. We have added the following text in the discussion to clarify this (P21, L45) “It has recently been shown that HLA-A11…”. Additionally we have added in the Liu et al (reference number 30).

2. At the beginning of the results, the characteristics of the 721.174 cell line (TAP-deficient) should be better explained to orient the reader as to how this cell line permits the peptide loading of HLA-C.

We have added in an additional sentence to clarify this (P15 Paragraph 1)

European Journal of Immunology

3. Equation (2) appears to be duplicated.

We have amended this

4. The histograms in figure 1 could be reduced in size, whereas the plots should be enlarged to increase resolution.

We have changed Figure 1 do this, but also added in the gating strategy as recommended in the editorial guidelines.

Reviewer: 3

Comments to the Author
The paper by Cassidy et al argues that there are differences in peptide selectivity between KIR2DL2 and KIR2DL3 with NK cells expressing the latter being more responsive to changes in peptide repertoire than those expressing 2DL2. While the data are broadly consistent with this hypothesis, the experimental system used to show this is extremely artificial and quite limited. Indeed, the paper relies entirely on responses to 3 closely related peptides, which may or may not be representative of different classes of endogenous peptides.

We agree this is an artificial system but it is one, which we have found to be informative in the context of group 1 specific HLA-C. It is known that KIR2DL2 and KIR2DL3 have different affinities for HLA-C but to date no functional differences have been noted. Our work demonstrates how this difference in affinity translates into a functional context. The presence of these receptors on different KIR haplotypes, may also be significant as KIR2DL2 is in tight linkage disequilibrium with KIR2DS2, but KIR2DL3 is not found with KIR2DS2. Model systems can be informative eg the use of the SIINFEKL peptide for T cells, and whilst we agree with this reviewers that this is a model system we think it illustrates a new aspect of NK cell biology, with potential implications for the association of KIR with the outcome of infection. We have amended the Discussion (P21, Paragraph 3) to reflect the comments of this reviewer "Our data show that in a model system changes in peptide repertoire lead more readily to activation of KIR2DL3…. and added in subsequently " Furthermore the in vivo significance of this work needs further exploration.." (see response to Reviewer 1 and 4).

The work itself looks to be technically quite tight – the assays are internally consistent and replicates are very close. Indeed the data appear to assign statistical significance to data that in magnitude looks to be barely different (eg Figs 2B, F)

European Journal of Immunology

We note this concern (see also response to Reviewer 1). We plotted the mean from each donor group and the SEM, hence the error bars are small. We have further discussed the analysis with a statistician and they have agreed that we have performed the appropriate statistical test ie an ANOVA with Student T-test corrected for multiple analyses. This takes into account both the overall differences for the 2DL2 and 2DL3 datasets (ANOVA), and also multiple analysis for comparisons of individual datapoints. This type of analysis is more rigorous than multiple T-tests, without correction, which are sometimes used to analyse this type of data. We therefore feel that the analysis is robust and reflects true differences between the donors.
Additionally in response to this reviewer and also Reviewer 1 we have redrawn Figs 2 and 3 to show individual donor points to display the primary data.

Fig 3 extends this analysis to more complex mixtures (3 peptides rather than 2) but the result is a slight broadening of the differences in response between 2Dl2+ and 2DL3+ NK cells, the data being broadly consistent with the idea that 2DL2 interacts with C1 with higher affinity than 2DL3 but nevertheless the differences are still modest with perhaps the 80% mix of VAPWNSRAL being the one exception where the differences are clearly appreciable. Moreover the authors provide an argument for why this context might be one in which the impact of VAPMWNSDAL on inhibition is more marked.

It's not clear the mathematical modeling reveals anything that the primary data themselves do not.

We note the reviewer's concern with the utility of this analysis. However we feel that its value lies in showing that in the mix of peptides it is the VAP-RA peptide alone rather than the VAP-DA: VAP-RA combination that is the most discriminatory between the two donors subsets and this analysis also confirms that VAP-RA is more discriminatory than VAP-FA. Additionally it does provide the first modeling of how NK cells may responds to changes in the peptide repertoire and Reviewer 4 notes it as a novel aspect of the study. Furthermore we feel that it provides a reference point for other researchers to test how NK cells may respond to changes in peptide repertoire. To address these issues we have added the following sentences to the discussion
"In the triple mix experiments the ratio of VAP-RA to VAP-DA appeared to be the most discriminatory between the donors, but in the mathematical analysis the concentration of VAP-RA appeared dominant over the combination of the two." (P21 Paragraph 1)
"The modeling data provide a template for how the inhibition of NK cells may change in response to the peptide content of MHC class I." (P22, L32)

I would think the analysis needs broadening to include a number of additional peptides to be comfortable that the conclusions are sound.

European Journal of Immunology

We agree that additional peptide combination would be helpful in this context and have therefore expended considerable effort in trying to provide this. We have generated a 721.221:HLA-C*0304 transfectant which has been further transfected with ICP47 in order to block TAP activity and facilitate peptide loading. We have tested GAVDPLLAL and its derivatives as described in Boyington et al Nature 200:405, 537-543. This set of peptide derivatives with P8 mutations has well described affinities (9.5, 42.3, 525 and >600μM) for KIR2DL2. However in our experiments none were antagonist. We therefore tested peptides P7 derivatives GAVDPLDAL, GAVDPLFAL, GAVDPLRAL. We found that GAVDPLDAL is antagonistic however GAVDPLRAL conferred greater inhibition than GAVDPLFAL. Therefore we feel that these differences require additional investigation before they are robust enough to present for publication, and the data ask additional questions as to the nature of an antagonist peptide. However we consider that the main point of the manuscript was not to investigate the mechanism of peptide antagonism but to demonstrate that there are differences in inhibition between KIR2DL2 and KIR2DL3 donors. We feel that we have demonstrated this in the best available system and that the additional studies to understand the subtle differences between the HLA-C*01 and HLA-C*03 systems require more in-depth investigation which are out of the scope of this current manuscript. We have therefore added a comment in the Discussion (P21, L34) "Testing of these data with additional peptide:HLA combinations would assist in establishing the broad applicability of these findings…"

Reviewer: 4

Comments to the Author
Peptide selectivity discriminates NK cells from KIR2DL2- and KIR2DL3-positive individuals.

In a continuation of their previous work (Fadda et al. PNAS 2010), the manuscript by Cassidy et al describes the results of a peptide competition assay in the context of NK cell degranulation. The authors have continued to use a model system comprised of three peptides and have extrapolated those findings to make general comments on the role of peptide repertoire on the activation of KIR2DL2 vs KIR2DL3 positive NK cells. The major concern regarding this paper is the level of conceptual advance made in comparison to their previous work (Fadda et al., PNAS 2010) – indeed, the current paper rests heavily on this prior work. To exemplify, the 2010 PNAS paper states in the discussion:

"KIR2DL3-positive NK cells may therefore be sensitive to a wider spectrum of changes in peptide repertoire than KIR2DL2-positive NK cells as a greater fraction of peptides could be antagonistic. This may in part explain the genetic association of KIR2DL3, but not KIR2DL2, with protection from hepatitis C infection"

European Journal of Immunology

While the abstract of this manuscript states: "KIR2DL3 positive NK cells were more sensitive to changes in the peptide content of MHC class I than KIR2DL2-positive NK cells." A major discriminating factor between the two pieces of work appears to be the mathematical modeling applied to this paper, and this may not be considered sufficiently suitable to warrant publication in EJI.

We note this reviewer's concerns. We agree that our work rests heavily on our previous work, in which we defined the phenomenon of peptide antagonism using our model system. However we feel that the work we now present has relevant and novel data. The major feature of this current work is that changes in peptide repertoire discriminate between KIR2DL2 and KIR2DL3 receptors. We did not demonstrate this in our PNAS paper, but we did speculate that this may be the case. Reviewer 4 highlights a sentence "KIR2DL3-positive NK cells may therefore be sensitive to a wider spectrum …" which we wrote in the Discussion section, rather than the Results section. This was an inference of the data we had generated, rather than based on our experimental data. We now provide the experimental data to support our hypothesis that KIR2DL2 and KIR2DL3 have different responses to changes in the peptide content of MHC class I. Additionally in the PNAS paper we only tested one donor with a KIR2DL2 homozygous genotype against the FA peptide alone and no KIR2DL2 homozygous donors against the FA:DA peptide combination, nor did we test the RA peptide in combination with other peptides in this PNAS paper. Thus we did not observe any significant donor differences in these limited experiments. However in this new manuscript, driven by differences in disease outcome related to donor KIR genotype, we have incorporated the RA peptide into peptide mixtures. The use of the RA peptide in the mixes is the key new feature that has allowed us to distinguish between the two different genotypes of donors.

Some general points:

1) It is not clear whether the DA peptide is a low affinity antagonistic peptide or a no-affinity peptide. Further to this, there appear to be differences in affinity for the RA peptide between KIR2DL2 and KIR2DL3 (Figure 1). This could go someway to explaining differences observed between these KIR2DLs. Precise details about the binding affinity would strengthen the paper.

Our previous work showed that DA is a low affinity antagonist peptide in that it induces clustering of KIR2DL3 at the immune synapse and also recruitment of SHP1 (Bohris et al J Immunol 2013:190;2924). However we agree that affinity measurements would give additional insights. It is well known that KIR2DL2 and KIR2DL3 have different affinities for HLA-C and this is related to Residues 16 and 148 (Moesta et al J Immunol 2008:180;3969-79). Both FA and RA induce lower levels of KIR2DL3 than KIR2DL2 binding, to HLA-C*0102 (Fadda et al., PNAS 2010: 107;10160). In order to gain some insights into this we have performed Scatchard analysis using the KIR-Fc constructs. This shows that the affinity of KIR2DL2 for both the FA and RA peptides is approximately twice that of KIR2DL3 (See Figure below).

European Journal of Immunology

Scatchard analysis of KIR2DL2-Fc and KIR2DL3-Fc binding to HLA-Cw*0102 and the indicated peptides. 721.174 cells were loaded with the indicated peptides at 10μM and then increasing concentrations of KIR-Fc were added. The binding was analysed by flow cytometry and plotted as a Scatchard plot. The slopes of the lines as determined by non-linear regression (GraphPad Prism 6) gives an approximation of binding affinity for each KIR.

Please note that the DA peptide did not inducing any measurable binding to either KIR2DL2 or KIR2DL3 KIR-Fc constructs. Therefore from this analysis, and from our studies on C*03 using the GAVPDLLAL peptide (see response to Reviewer 3), it is not clear that it is purely the relative affinities that determine the scope for peptide antagonism and the differences between the two donors. The mechanisms for our observations currently require further investigation, in more detailed structural studies which we feel is out of the scope of this current manuscript, the main aim of which was to determine if there were observable differences in the inhibitory potential of NK cells from KIR2DL2 and KIR2DL3 homozygous donors.

2) A confounding variable that has not been addressed is the allele of KIR2DL that is carried by the donors. Previous studies have observed that KIR2DL polymorphism can significantly impact on HLA-C recognition and signalling. What is presented in this study is a mean and SEM for a number of donors. It would be interesting to see the distribution by plotting all donors. Further, if the alleles of the donors are known then they should be presented.

We note this point and have reorganised the data in Figs 2 and 3 to address this and also that of Reviewer 1. Unfortunately we did not perform allelic level typing in these individuals. The more frequent KIR2DL2 and KIR2DL3 alleles in the Caucasian populations (KIR2DL2*001, KIR2DL2*003, KIR2DL3*001 and KIR2DL3*002 [http://www.allelefrequencies.net/kir6002a.asp]) differ by the same three differences in the ligand binding D1 and D2 domains including residues 16 and 148, which are responsible for the affinity differences between KIR2DL2 and KIR2DL3. The fourth difference in the D1 and D2 domains is an isoleucine:threonine polymorphism at residue 200. 2DL2*001 has isoleucine, but KIR2DL2*003, KIR2DL3*001 and KIR2DL3*002 all have threonine. Therefore overall we feel that typing to the allelic level is unlikely to have a substantial impact on our findings.

3) Figure 1A provides evidence that all the peptides bind HLA-C. However, can the authors comment on the relative affinity of the peptides for HLA-C? If the DA peptide has a higher affinity than the other peptides then there will be more on the cell surface than the ratios added would suggest.

We have used peptide stabilization of HLA-C*0102 on 721.174 cells to determine the affinity of the peptides for HLA-C. These show that the affinities are similar, with similar dissociation constants (VAPWNSFAL Kd=0.14μM, VAPWNSRAL Kd=0.19μM and VAPWNSDAL Kd=0.14μM with the wild-type peptide VAPWNSLSL Kd=0.18μM). We have added this data as a supplementary figure (Supporting information Fig 1) and referenced it in the Results section (P15, L27). Additionally in our previous manuscript (Faddda et al PNAS 2010) we had performed assays of peptide antagonism at a final concentration of one tenth saturating levels of peptide and had similar results, implying that peptide displacement is not a major mechanism for peptide antagonism.

4) Discussion: Paragraph 3:

The line " The implications of these data are that changes in peptide repertoire lead more readily to activation of KIR2DL3… than KIR2DL2 positive NK cells". This statement is perhaps too strong and should be limited to the model system that was used. Especially when considered in the absence of affinity data, the absence of the alleles of KIR2D being presented and the limitations of the study to just three peptides.

We note this concern and have amended this to "Our data show that in a model system changes in peptide repertoire lead more readily to activation of KIR2DL3…. and added in subsequently "Furthermore the in vivo significance of this work needs further exploration.." (see also response to Reviewer 1) (P21 paragraph 3).

Peer review correspondence

Second Editorial Decision – 19 September 2014

Dear Dr. Khakoo,

It is a pleasure to provisionally accept your manuscript entitled "Peptide selectivity discriminates NK cells from KIR2DL2- and KIR2DL3-positive individuals" for publication in the European Journal of Immunology.

For final acceptance, please follow the instructions below and return the requested items as soon as possible as we cannot process your manuscript further until all items listed below are dealt with.

Please note that EJI articles are now published online a few days after final acceptance (see Accepted Articles: http://onlinelibrary.wiley.com/journal/10.1002/(ISSN)1521-4141/accepted). The files used for the Accepted Articles are the final files and information supplied by you in Manuscript Central. You should therefore check that all the information (including author names) is correct as changes will NOT be permitted until the proofs stage.

We look forward to hearing from you and thank you for submitting your manuscript to the European Journal of Immunology.

Yours sincerely,
Laura Soto Vazquez

on behalf of
Prof. Marco Colonna

Editorial Office
European Journal of Immunology
e-mail: ejied@wiley.com
www.eji-journal.eu
